# Strengthening of Traditional Chinese Medicine in the Health System Reform: Effect on Health Outcomes and Financial Protection

**DOI:** 10.1155/2022/7226674

**Published:** 2022-01-19

**Authors:** Ping He, Dawei Zhu, Xiaowei Man, Qian Bai, Lieyu Huang, Xuefeng Shi, Qingyue Meng

**Affiliations:** ^1^China Center for Health Development Studies, Peking University, Beijing 100191, China; ^2^School of Management, Beijing University of Chinese Medicine, Beijing 100029, China; ^3^State Key Laboratory of Quality Research in Chinese Medicine, Institute of Chinese Medical Sciences, University of Macau, Macau, Taipa 999078, China; ^4^Office of Policy and Planning Research, Chinese Center for Disease Control and Prevention, Beijing 102206, China

## Abstract

**Background:**

The challenges of modern medicine in addressing chronic diseases necessitate a shift of attention towards traditional medicine (TM) and other supplementary care systems. China has prioritized the strengthening of traditional Chinese medicine (TCM) in the health system reform since 2009. This study sought to assess the effects of the reform on TCM and the resultant effect of a strengthened TCM on health outcomes and financial protection.

**Methods:**

Longitudinal data were obtained from the China Statistical Yearbook, China Health Statistical Yearbook, China Population Statistical Yearbook, and Statistical Extract of Traditional Chinese Medicine in 31 provinces of mainland China between 2002 and 2016. Dependent variables included health outcomes measured by age-standardized excess mortality and life expectancy at birth and financial protection measured by the proportion of health expenses in total consumption expenses. The independent variables consisted of the number and proportion of TCM physicians. The fixed effects (FEs) models were established to identify the effect of the independent variables on outcomes.

**Results:**

From 2009 to 2016, the number and proportion of TCM physicians increased from 22 to 36 physicians per 100,000 population and from <12% to >15%, respectively. The changes were more rapid and higher than that in the period before the reform. An increase of 1 TCM physician per 100,000 population was associated with a decrease of 1.944 excess deaths, a 5.84-day increase in male life expectancy, and a decrease of 0.051% of health expenses among both urban and rural residents. An increase in proportion of 1% of TCM physicians was associated with a decrease of 5.097 excess deaths, a 17.52-day increase of life expectancy (both genders), an increase of 21.535-day in life expectancy (males) per 100,000 population, and a decrease of 0.082% of health expenses among rural residents.

**Conclusion:**

During China's health system reform, the increased physician number has strengthened TCM. Higher TCM physician supply was associated with improved health outcomes and financial protection, which implies that the reform may have important implications on health system performance in China.

## 1. Background

The crisis of modern medicine, manifested by the inability to cure major chronic diseases, such as cancer and heart disease, has aroused international communities to recognize importance to the use of traditional medicine (TM) [1]. In the process of finding cure, modern science has developed multiple medications for treating these diseases. However, most of them are not affordable and have concomitant side effects and morbidities [2]. In addition, chronic diseases have replaced infectious diseases as the major cause of disease and healthcare-related financial burden in both developed and developing countries. During the past two decades, China's health profile has rapidly changed towards a health system mainly burdened by multiple chronic diseases, including cardiovascular diseases, cancers, and mental disorders [3]. In addressing these health challenges, western medicine has shown its limitations in providing effective preventive and curative care and the need to explore more intensive use of alternative medicine.

This concern repositions TM as an option in mainstream medicine to solve future health problems in several countries and regions, especially for those with rapidly ageing populations. The first TM strategy report, “WHO Traditional Medicine Strategy 2002–2005”, and the updated version, “WHO Traditional Medicine Strategy 2014–2023”, stressed the significance of TM and appealed to the whole world to adopt appropriate policies to support the development of TM [4]. The WHO strategies have played a key role in integrating TM into modern medical systems, especially in low-resource countries where most of the population rely on TM. For example, in Africa, by 2012, 40 countries had national TM policies, and 39 countries had established national TM offices, as compared with 8 and 15 countries in 2000, respectively [5].

China has a long history of TM and a cultural heritage of traditional Chinese medicine (TCM), dating back to over 2000 years. TCM, which includes herbal medicine, acupuncture, moxibustion, massage, food therapy, and physical exercise [6], has contributed significantly to the country's procreation, development, and human civilization [7]. Facing similar challenges of modern medicine as western societies, China is rethinking the essence and contributions of TCM and attempt to rebuild a holistic medical system to integrate TCM and western medicine. For example, the Chinese Academy of Engineering is organizing an ambitious national project to conduct studies on the strategy of holistic medicine 2035, including the consolidation of TCM and western medicine.

High-level Chinese policy makers have interest in TCM. During China's health system reform launched in 2009 with the goal of providing equitable and affordable essential health services for all [8], the government focused on strengthening TCM by implementing the improvement project of TCM service ability in 2012, enacting the first TCM law in 2016, and designing a strategic plan for TCM (2016–2030). The national policies and law explicitly emphasized that strengthening human resource is the major key to the development of TCM. Specifically, in 2012, the Chinese government stated that 95% of community health centers, 90% of township health centers, 70% of community health stations, and 65% of healthcare clinics should be able to provide TCM services by 2015, requiring substantial growth in the number of TCM physicians, relative to the total physician population.

The Lancet, in its first series on China's health system reform in 2008, indicated that TCM was effective for health outcomes, such as pain and survival. A decade after The Lancet made this statement, the time is right to examine the statement with evidence [9]. In addition, many reports on disease burden in the world and China have been published in The Lancet and other sources. More interestingly, policy makers would learn more about how disease burden could be effectively addressed by strengthening the existing health system [10, 11]. Lack of evidence on whether TCM is beneficial to population-based health outcomes discourages belief and support for TCM. Using nationwide databases, this study aimed to examine (1) if the reform effectively strengthened TCM and (2) if strengthening of TCM has had positive effects on health outcomes and financial protection. This study proposes to improve our understanding of the effect of TCM. The findings have implications for the performance evaluation of the input of TCM.

## 2. Methods

### 2.1. Data Sources

This study analyzed longitudinal data of 31 regions (simplified as provinces) in mainland China during the period of 2002 to 2016. The unit of analysis was each province at each year (province-year). To obtain the variables of interest, data from several statistical databases were selected and combined into a unique data set. The data were principally obtained from four official sets of 15-year statistical yearbooks, which included the China Statistical Yearbook, China Health Statistical Yearbook, China Population Statistical Yearbook, and Statistical Extract of Traditional Chinese Medicine.

### 2.2. Measures

Based on the goals of a health system identified by the WHO Health Report 2000 [12], our outcomes included two types of measures: population health and financial protection. Population health was measured by age-standardized excess mortality and life expectancy at birth (for all genders combined and for each gender separately). Age-standardized excess mortality is the difference between the mortality observed in a province and the mortality that would have occurred in that province if it had the same mortality as the nation. This is represented as equation (1).

(1) $$\begin{array}{c} {\text{ASEM}}_{it}={\text{OM}}_{it}-\sum_{j}^{k}P_{itj}\times {\text{NM}}_{tj}, \end{array}$$

where ASEM_*it*_ denotes the age-standardized excess mortality in province *i* at year *t*; OM_*it*_ is the observed mortality in province *i* at year *t*; *P*_*itj*_ denotes the proportion of age group *j* in province *i* at year *t*; and NM_*tj*_ represents the national mortality in age group *j* at year *t*. Financial protection was measured by health expenses as percentages of total consumption expenses (among urban and rural residents).

The study identified two separate independent variables, including the number of TCM physicians per 100,000 population and the proportion of TCM physicians among all physicians, to measure the absolute and relative resource of TCM, respectively.

Covariates capable of confounding the association between the resource of TCM and outcomes at the population level included dependency ratio (proportion of population under 15 years and over 65 years), illiteracy rate in the population above 15 years, and GDP per capita. Dependency ratio was used to represent the population age structure. GDP per capita was used to control for economic development differences among the provinces and yearly price differences. The illiteracy rate reflected overall educational status in each province.

### 2.3. Statistical Analysis

This study used a longitudinal data set to assess the relationships between the dependent and independent variables over a 15-year period. To offset potential problems associated with omitted variable biases, the fixed effects (FEs) model was applied. An advantage of the FE model is that it controls for time-invariant heterogeneity among provinces, such as underlying aspects of local culture. To control for recent health reforms, time dummies were included to control aggregate time-specific effects that may affect outcomes, which would not be considered by province explanatory variables. Logarithmic transformations were performed on per capita GDP and illiteracy rate to adjust for the skewed monetary variables. Missing data on life expectancy at birth (for all genders combined and for each gender separately) were imputed by linear interpolation (extrapolation).

Three robust tests were used to check our results. First, the independent variable was replaced with the proportion of TCM hospitals and TCM hospital beds. Second, the dependent variables were replaced with crude mortality per 100,000 population and health expenses as a percentage of total income (among urban and rural residents). Third, the study classified the 31 provinces into two groups by healthcare resource and examined whether the association varied across regions with various healthcare resources.

A *p* value of <0.05 was considered statistically significant. Stata version 15 for Windows (Stata Corp, College Station, TX, USA) was used for all statistical analyses.

## 3. Results

### 3.1. Sample Characteristics

Table 1 shows the characteristics of the study sample. Crude mortality decreased by 19.61 per 100,000 population during 2002 to 2009 and increased by 19.29 per 100,000 population during 2009 to 2016. Life expectancy at birth gradually increased in the total population, with an increment of 2.56 years during 2002 to 2009 and 2.56 years during 2009 to 2016. Life expectancy at birth in females, which was higher than that in males in 2002, increased more rapidly than males during both 2002 to 2009 and 2009 to 2016. Among urban residents, the proportion of health expenses in total consumption expenses increased by 0.04% during 2002 to 2009 and by 0.28% during 2009 to 2016. Among rural residents, the proportion of health expenses in total consumption expenses increased by 1.58% and 1.84% during 2002 to 2009 and 2009 to 2016, respectively. The number of TCM physicians slightly increased by 0.17 per 100,000 persons during 2002 to 2009, and rapidly increased by 13.98% during 2009 to 2016. The proportion of TCM physicians among all physicians decreased by 1.73% during 2002 to 2009 and increased by 3.50% during 2009 to 2016. The characteristics of the control variables are shown in Table 1.

### 3.2. Changes in the Number and Proportion of TCM Physicians before and after the Health System Reform

**Figure 1:**
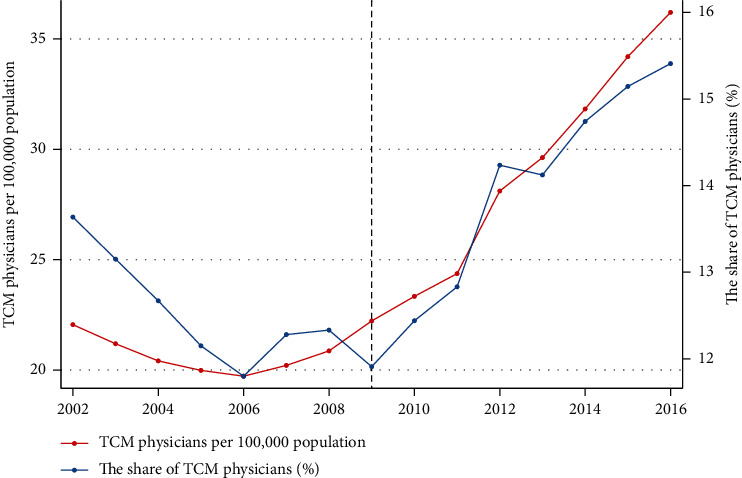
Year-by-year changes in number and proportion of TCM physicians per 100,000 population, 2002 to 2016.

Figure 1 presents yearly changes in the number and proportion of TCM physicians in China. In the prereform period (2002–2009), the number of TCM physicians fluctuated and reached its minimum in 2006. In the postreform period (2009–2016), the number increased rapidly. In addition, before 2009, the proportion of TCM physicians relative to all physicians also fluctuated and reached its minimum in 2006. After 2009, this figure increased to approximately 16% in 2016.

**Figure 2:**
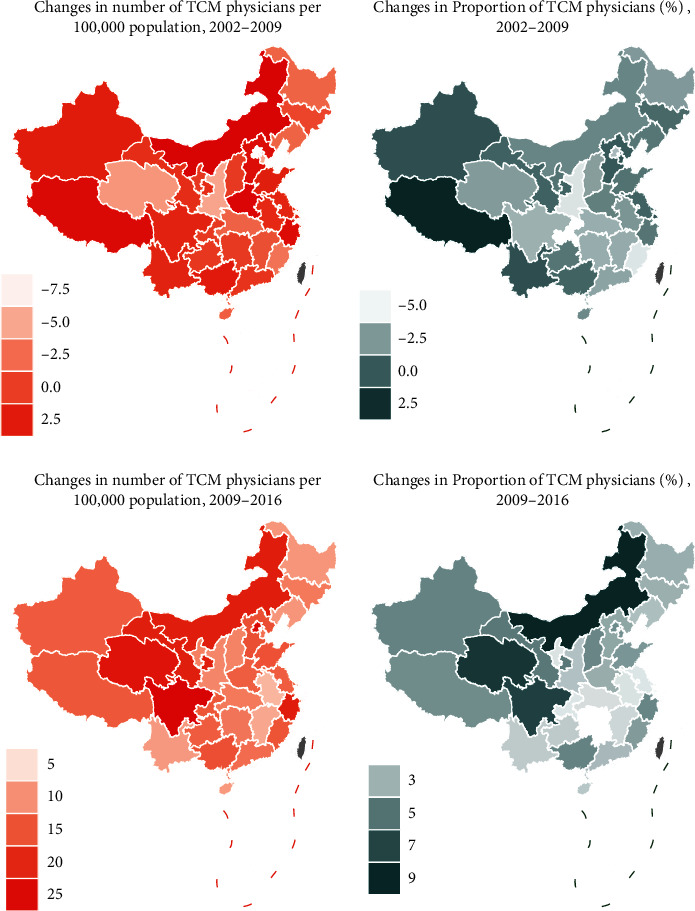
Changes in number and proportion of TCM physicians per 100,000 population in 31 provinces of China.

Figure 2 illustrates changes in the number and proportion of TCM physicians by provinces before and after the 2009 health system reform. In the prereform period, only few provinces experienced an increase in the number and proportion of TCM physicians. In the postreform period, an increasing number of provinces experienced an increase in number and proportion of TCM physicians.

### 3.3. The Effect of Changes in TCM Physicians on Health Outcomes and Financial Protection

Table 2 shows the FE regression results on the effect of TCM physicians on multiple health outcomes and financial protection. Model 1 used the number of TCM physicians as the independent variable. The model revealed that an increase of 1 TCM physician per 100,000 population was associated with a decrease of 1.944 excess deaths, a 5.84-day increase in male life expectancy, and a decrease of 0.051% of health expenses in total consumption expenses in both urban and rural residents.

Using the proportion of TCM physicians as the independent variable, Model 2 revealed that an increase of 1% of TCM physicians among total physicians was associated with a decrease of 5.097 excess deaths, a 17.52-day increase in life expectancy (both genders), a 21.535-day increase in life expectancy (males) per 100,000 population, and a decrease of 0.082% of health expenses in total consumption expenses among rural residents (Table 2).

### 3.4. Robustness Check

Multiple ways were used to check the robustness of the FE regression model regarding the effect of TCM physicians (Table 3). First, the proportion of TCM hospitals and hospital beds was used as an alternative independent variable to check for changes associated with health outcomes and financial protection (Model 3 and 4). Significant associations were observed between increased proportion of TCM hospitals and hospital beds and improved life expectancy (all genders, females, and males) and decreased health expenses in total consumption expenses among rural residents.

Second, crude mortality was used as an alternative measure of health outcomes and proportion of health expenses of total income as an alternative indicator of financial protection. An association was observed between increased number of TCM physicians and decreased proportion of health expenses in total consumption expenses among urban residents (Model 5). In addition, increased proportion of TCM physicians was related to decreased crude mortality (Model 6).

Third, to evaluate the heterogeneity of health resources on our regressions, FE regression was analyzed by level of health resources among the provinces. The number and proportion of TCM physicians among all physicians were each associated with some health outcomes and financial protection variables (Model 7 and 8).

## 4. Discussion

TCM physician supply in terms of both absolute and relative number significantly increased after 2009, which marked the beginning of China's health system reform. Higher number and proportion of TCM physicians were associated with lower excess mortality, higher aggregate and gender-specific life expectancy, and lower proportion of health expenses in total consumption expenses. These observations imply that the strengthening of the TCM system will most likely contribute significantly to population-based health outcomes and improve financial protection.

The study findings revealed that TCM physician supply experienced a substantial growth after the 2009 health system reform. Different from TM in western societies [13], TCM has been promoted to be a major component of China's healthcare system since the early 1950s [14]. However, following China's reform and opening-up policy in the late 1970s, the cultural importance of TCM waned due to the profit-seeking behavior of healthcare facilities, resulting in decreased patronization of TCM [15]. In the 1990s, some studies found a downward trend in the number of TCM physicians at TCM hospitals [16]. Our study also observed a decline in the amount of TCM physicians per 100,000 population from 2002 to 2009. Afterwards, during 2009 to 2016, TCM physician supply increased substantially. As no significant changes have occurred in policies on TCM human resources, except the health system reform, we speculate that the increased number of TCM physicians is due to the 2009 reform.

At the province level, associations were observed between TCM physician supply and health system performance, which underscores the importance of supporting TCM development. Prioritization of TCM development has been a controversial issue during the past decades. One argument focuses on the effectiveness of TCM. A study based on 70 Cochrane systematic reviews concluded that most studies on the efficacy of TCM were inconclusive, while others reported preliminary benefits to certain patient populations [17]. In this study, using provincial data, we observed that the higher number and proportion of TCM physicians were associated with improved health outcomes, in terms of lower age-standardized excess mortality and higher aggregate and gender-specific life expectancy. This observation is likely due to a number of reasons.

Theoretically, TCM treatment is beneficial to population health. Guided by TCM treatment principles, such as treating the root of disease, reinforcing the healthy Qi (Zheng Qi), eliminating the evil Qi (Xie Qi), and harmonizing Yin and Yang, the body recovers spontaneously by means of arousing and motivating the body's self-regulating function. TCM cures disease by improving immunity function and strengthening resistance to disease, which means the internal healthy Qi can prevent the body from pathogenic factors [18, 19].

According to TCM, the human body is a holistic system, which indicates that the constituents of the human body, including organs and tissues, are indivisible in structure, coordinate in physiology, and affect each other in pathology [20]. External factors, involving geographical location, natural environment, and climatic variations, could affect the human body both in physiology and pathology. TCM emphasizes the unity and coordination among the components of the human body, as well as the harmony between the human body and its external surroundings [21]. Under the guidance of holism, TCM forms its unique basic theory, which further contributes to the construction of human-life science, physiological, pathological, and treatment theories with distinctive TCM features. Furthermore, previous studies have proven that TCM treatments were beneficial for patients with cardiovascular and cerebrovascular diseases [22], cancer [23, 24], and chronic obstructive pulmonary disease (COPD) [25], which were three leading causes of deaths in the Chinese population [3].

In addition, our findings showed that the larger number and proportion of TCM physicians were associated with a lower proportion of resident health expenses in total consumption expenses, suggesting that TCM physician supply had potential benefits for financial protection. In treating some health problems, TCM has the relative advantage of lower cost over western medicine. For example, economic evaluations showed that healthcare costs from use of TCM only was far lower than that of western medicine only, in treating the same diseases [26] or having the same curative effects [27] at primary-care facilities. Our study further confirmed this finding at the population level.

This study had several limitations. First, given the association between TCM physician supply and health performance outcomes at the population level, our analyses were appropriately ecological. This limits the extent to which causal associations between TCM development and health system performance can be established. Second, the study failed to provide evidence on which part of the TCM system was most effective for population health and financial protection, although the number and proportion of TCM physicians were beneficial in determining the overall contribution of TCM. Despite the limitations, to the best of our knowledge, this was the first study to investigate the effect of TCM development on health system performance using multiple outcomes and considering several confounders.

## 5. Conclusion

The increased TCM physician supply was associated with improved health outcomes and financial protection. The significant increase in TCM physician supply after China's health system reform in 2009 implies that strengthening of TCM is an effective strategy for healthy China. Further studies are needed to examine the causal relations between TCM and health when more data are available.

## Figures and Tables

**Table 1:** Characteristics of the study sample in 31 provinces of China, 2002 to 2016.

	Mean (95% CI)				
	2002	2009	2016	Within-province change	
				2002 to 2009	2009 to 2016
Outcome variables					
Crude mortality per 100,000 population	606.97 (585.12, 628.81)	587.35 (562.15, 612.56)	606.65 (576.13, 637.16)	−19.61 (−37.70, −1.52)	19.29 (−3.43, 42.01)
Age-standardized excess mortality per 100,000 population	−22.42 (−58.25, 13.40)	62.07 (32.21, 91.94)	94.72 (66.13, 123.31)	−	−
Life expectancy at birth (both genders)	71.98 (70.84, 73.11)	74.54 (73.52, 75.56)	77.10 (76.16, 78.04)	2.56 (2.36, 2.77)	2.56 (2.36, 2.77)
Life expectancy at birth (female)	74.01 (72.80, 75.22)	77.05 (76.01, 78.09)	80.09 (79.15, 81.02)	3.04 (2.73, 3.35)	3.04 (2.73, 3.35)
Life expectancy at birth (male)	70.13 (69.06, 71.20)	72.32 (71.29, 73.34)	74.50 (73.51, 75.50)	2.19 (2.02, 2.35)	2.19 (2.02, 2.35)
Health expenses as a percentage of total consumption expenses among urban residents (%)	7.19 (6.61, 7.77)	7.23 (6.62, 7.85)	7.51 (6.82, 8.21)	0.04 (−0.28, 0.37)	0.28 (−0.11, 0.68)
Health expenditures as a percentage of total consumption expenses among rural residents (%)	5.90 (5.28, 6.52)	7.48 (6.65, 8.31)	9.32 (8.45, 10.20)	1.58 (1.05, 2.11)	1.84 (1.22, 2.46)

Independent variables					
TCM physicians per 100,000 population	22.06 (18.48, 25.64)	22.22 (19.17, 25.28)	36.20 (31.88, 40.53)	0.17 (−0.96, 1.30)	13.98 (12.04, 15.92)
The proportion of TCM physicians (%)	13.64 (12.26, 15.01)	11.91 (10.81, 13.01)	15.41 (14.02, 16.80)	−1.73 (−2.40, −1.06)	3.50 (2.83, 4.17)

Control variables					
Dependency ratio (%)	41.40 (39.08, 43.72)	35.95 (33.71, 38.19)	37.18 (35.12, 39.24)	−5.45 (−6.87, −4.03)	1.23 (0.16, 2.30)
Illiteracy rate (%)	12.90 (10.02, 15.79)	8.28 (5.76, 10.80)	6.46 (3.89, 9.02)	−4.62 (−5.46, −3.78)	−1.82 (−2.48, −1.17)
GDP per capita (thousand RMB)	10.32 (7.43, 13.21)	28.22 (22.51, 33.92)	56.77 (47.33, 66.20)	17.90 (14.68, 21.12)	28.55 (24.42, 32.68)

**Table 2:** Fixed Effects Regression Results on the effect of TCM Physicians on Health Outcomes and Financial Protection, 2002 to 2016.

	Model 1 (TCM physicians per 100,000 population)	Model 2 (proportion of TCM physicians, %)
Health outcomes		
Age-standardized excess mortality per 100,000 population	−1.944 (−3.269, −0.619)^∗∗^	−5.097 (−8.092, −2.102)^∗∗∗^
Life expectancy at birth (both genders/days)	2.555 (−1.737, 6.847)	17.520 (7.504, 27.536)^∗∗∗^
Life expectancy at birth (female/days)	−2.920 (−9.359, 3.519)	10.220 (−4.803, 25.243)
Life expectancy at birth (male/days)	5.840 (2.263, 9.417)^∗∗^	21.535 (12.950, 30.120)^∗∗∗^

Financial protection		
Health expenses as a percentage of total consumption expenses among urban residents (%)	−0.051 (−0.076, −0.026)^∗∗∗^	−0.044 (−0.101, 0.013)
Health expenses as a percentage of total consumption expenses among rural residents (%)	−0.051 (−0.086, −0.016)^∗∗^	−0.082 (−0.162, −0.002)^*∗*^

The model was adjusted for province and yearly fixed effects, dependency ratio (%), illiteracy rate (%), and GDP per capita (thousands RMB). 95% CI in parentheses. ^∗∗∗^*p* < 0.001,^∗∗^*p* < 0.01,^*∗*^*p* < 0.05.

**Table 3:** Robustness check.

	Model 3 (proportion of TCM hospitals, %)		Model 4 (proportion of TCM hospital beds, %)	
Health outcomes				
Age-standardized excess mortality per 100,000 population	−0.004 (−1.907, 1.899)		−1.178 (−4.077, 1.721)	
Life expectancy at birth (both genders/days)	16.425 (10.702, 22.148)^∗∗∗^		30.295 (20.995, 39.595)^∗∗∗^	
Life expectancy at birth (female/days)	12.775 (3.475, 22.075)^∗∗^		34.675 (21.082, 48.268)^∗∗∗^	
Life expectancy at birth (male/days)	19.345 (14.337, 24.353)^∗∗∗^		25.915 (18.046, 33.784)^∗∗∗^	

Financial protection				
Health expenses as a percentage of total consumption expenses among urban residents (%)	−0.016 (−0.051, 0.019)		−0.046 (−0.101, 0.009)	
Health expenses as a percentage of total consumption expenses among rural residents (%)	−0.138 (−0.187, −0.089)^∗∗∗^		−0.102 (−0.178, −0.026)^*∗*^	
	Model 5 (number of TCM physicians per 100,000 population)		Model 6 (proportion of TCM physicians, %)	

Health outcomes				
Crude mortality per 100,000 population	−1.107 (−2.25, 0.036)		−6.715 (−9.224, −4.206) ^∗∗∗^	

Financial protection				
Health expenses as a percentage of total income among urban residents (%)	−0.027 (−0.047, −0.007) ^∗∗^		−0.023 (−0.062, 0.016)	
Health expenses as a percentage of total income among rural residents (%)	0.018 (−0.013, 0.049)		0.061 (0.002, 0.12)	

	Model 7 (number of TCM physicians per 100,000 population)		Model 8 (proportion of TCM physicians, %)	
	Low-health resource provinces	High-health resource provinces	Low-health resource provinces	High-health resource provinces

Health outcomes				
Age-standardized excess mortality	−1.715 (−3.138, −0.292)^*∗*^	−1.934 (−3.261, −0.607)^∗∗^	−5.060 (−8.057, −2.063)^∗∗^	−5.490 (−8.610, −2.370)^∗∗∗^
Life expectancy at birth (both genders/days)	6.205 (1.913, 10.497)^∗∗^	2.555 (−1.737, 6.847)	17.885 (7.869, 27.901)^∗∗∗^	12.045 (2.029, 22.061)^*∗*^
Life expectancy at birth (female/days)	3.285 (−3.869, 10.439)	−2.555 (−8.994, 3.884)	10.950 (−3.358, 25.258)	1.460 (−13.563, 16.483)
Life expectancy at birth (male/days)	8.030 (3.738, 12.322)^∗∗∗^	6.205 (2.628, 9.782)^∗∗^	21.900 (13.315, 30.485)^∗∗∗^	18.250 (9.665, 26.835)^∗∗∗^

Financial protection				
Health expenses as a percentage of total consumption expenses among urban residents (%)	−0.048 (−0.073, −0.023)^∗∗∗^	−0.051 (−0.076, −0.026)^∗∗∗^	−0.042 (−0.099, 0.015)	−0.059 (−0.118, 0.000)
Health expenses as a percentage of total consumption expenses among rural residents (%)	−0.044 (−0.081, −0.007)^*∗*^	−0.050 (−0.085, −0.015)^∗∗^	−0.081 (−0.161, −0.001)	−0.095 (−0.179, −0.011)^*∗*^

Model was adjusted for province and yearly fixed effects, dependency ratio (%), illiteracy rate (%), and GDP per capita (thousands RMB). 95% CI in parentheses. ^∗∗∗^*p* < 0.001,^∗∗^*p* < 0.01,^*∗*^*p* < 0.05.

## Data Availability

The data are available from the China Statistical Yearbook, China Health Statistical Yearbook, China Population Statistical Yearbook, and Statistical Extract of Traditional Chinese Medicine.

## List of abbreviations

TM: Traditional medicine
TCM: Traditional Chinese medicine
FE: Fixed effects
COPD: Chronic obstructive pulmonary disease.
